# Multi-modal imaging and analysis in the search for iron-based magnetoreceptors in the honeybee *Apis mellifera*

**DOI:** 10.1098/rsos.181163

**Published:** 2018-09-19

**Authors:** Jeremy A. Shaw, Alastair Boyd, Michael House, Gary Cowin, Boris Baer

**Affiliations:** 1 Centre for Microscopy, Characterisation and Analysis, The University of Western Australia, Perth, Western Australia 6009, Australia; 2 School of Physics, The University of Western Australia, Perth, Western Australia 6009, Australia; 3 Centre for Advanced Imaging, The University of Queensland, Brisbane, Queensland 4072, Australia; 4 Centre for Integrative Bee Research, Department of Entomology, University of California, Riverside, CA 92521, USA

**Keywords:** magnetoreception, iron, honeybee

## Abstract

The honeybee *Apis mellifera* is one of many animal species for which empirical evidence of a magnetic sense has been provided. The underlying mechanisms postulated for magnetoreception in bees are varied, but most point towards the abdomen as the most likely anatomical region for its location, partly owing to the large accumulation of iron in trophocyte cells that comprise the honeybee fat body. Using a multi-modal imaging and analysis approach, we have investigated iron in the honeybee, with a particular focus on the abdomen and the utility of such techniques as applied to magnetoreception. Abdominal iron is shown to accumulate rapidly, reaching near maximum levels only 5 days after emerging from the comb and is associated with the accumulation of iron within the fat body. While fat body iron could be visualized, no regions of interest, other than perhaps the fat body itself, were identified as potential sites for magnetoreceptive cells. If an iron-based magnetoreceptor exists within the honeybee abdomen the large accumulation of iron in the fat body is likely to impede its discovery.

## 1. Introduction

Biogenic magnetite nanoparticles, such as those present in magnetotactic bacteria, form the basis of the magnetite hypothesis of magnetoreception, which postulates that specialized neuro-receptors containing nanoparticulate magnetite are responsible for the well-documented phenomenon of magnetic navigation in animals (see for example: [1]). However, the search for such particles in animals has proven to be extremely challenging and, despite more than 50 years of investigation, evidence for their existence remains elusive. A lack of knowledge about the morphological location of magnetoreceptors, together with the widespread occurrence of endogenous and exogenous sources of iron have proven to be major obstacles that have slowed progress towards the validation of the magnetite hypothesis [2]. It should be noted that while magnetite remains the most likely candidate mineral owing to its magnetic properties, less magnetic forms of iron oxide are being re-examined [3].

In the context of magnetoreception, iron contamination acts to mask or interfere with the process of identification and characterization of the true signature of iron material associated with a magnetoreceptor. Contaminating iron material is common; occurring naturally as a vital component of metabolism and is ubiquitous in both the environment and laboratory settings [4–6]. Recently, the issue of iron contamination has featured prominently in the literature, where cells presented as putative magnetoreceptor structures were later shown to have been misidentified. For almost a decade, magnetite-containing cells located at six specific loci in the upper beak of pigeons were thought to be responsible for their magnetic sensitivity [7,8]. These were subsequently shown to be iron-rich macrophage cells, not magnetoreceptive neurons [9]. In a separate study, a rotating magnetic field was used to identify putative magnetoreceptor cells from preparations of dissociated trout olfactory epithelia [10]. The magnetic behaviour of cells identified by this screening method was later shown to be caused by the attachment of contaminating substances [11]. These sets of studies are significant as they have emphasized the pitfalls associated with the presence of endogenous and exogenous iron and the need for a rigorous, correlative approach to imaging and analysis when attempting to characterize magnetoreceptor systems.

The European honeybee (*Apis mellifera*) is hypothesized to be a magnetoreceptive insect, as supported by behavioural studies [12–17], magnetometry-based analyses [18–21] and the direct observation of magnetic material in worker bees [22–25]. Specifically, the honeybee abdomen has been identified as a possible location for a magnetoreceptor system because of the presence of nanoscale magnetite particles [19,21]. Furthermore, magnetized wires attached to the dorsal abdomen of bees have been found to interfere with their ability to discriminate variations in magnetic fields, whereas wires attached to other body parts or non-magnetic control wires had no effect [17]. More recently, it was demonstrated that honeybees can be trained to elicit a proboscis extension reflex in response to magnetic field exposure, which is interrupted when the ventral nerve cord between the thorax and abdomen is severed [26]. A magnetoreceptor based on superparamagnetic magnetite was proposed to be present in hairs on the anterior dorsal region of the abdomen [27] but other studies have postulated the abdominal fat body as a target site because of the presence of iron granules [22,28,29]; but see [30]. In summary, the location and function of a magnetic receptor remains unresolved in honeybees, despite a substantial amount of work conducted on their magnetic sense.

Unravelling the cellular basis of an iron-based magnetoreceptor will first require the development of novel experimental approaches that can control contamination problems associated with the widespread occurrence of iron in organisms. Additionally, methods for defining regions of interest to address the ‘needle-in-a-haystack’ problem associated with searching without an anatomical target are needed [2]. In addition to the evidence supporting honeybees as a magnetoreceptive species, they represent an ideal model system for optimizing new experimental approaches. (i) They are small with a well-defined body plan that can be divided into discrete segments suitable for various imaging modalities, while reducing the volume of tissue in the search area. (ii) Large numbers of individuals can be collected for bulk analyses. (iii) Their eusocial behaviour and hive structure make various experimental manipulations and husbandry possible. The presence of large accumulations of iron in the honeybee abdomen also represents an opportunity to highlight further aspects of this iron store, in the context of either a magnetoreception system or as a source of endogenous iron contamination.

We have applied a range of analytical and imaging methods to characterize and study iron accumulation in different body parts of honeybee workers. To do this we used mass spectrometry to quantify iron levels in the antennae, head, thorax and abdomen of worker bees at various ages and used a combination of optical, electron, X-ray and magnetic resonance imaging methods. Such data serves to provide baseline information for further investigations into the biology of this economically important insect species, including determinations on the phenomenon of honeybee magnetoreception.

## 2. Material and methods

### 2.1. Animal collection

We used the honeybee *Apis mellifera* for all experimental work, which we sourced from hives kept at an apiary located at the University of Western Australia. To obtain newly eclosed bees (day zero), we removed brood frames from nucleus hives, removed all adult bees and placed the frames into an incubator at 32°C. Eclosing bees were brushed from the frame and either used immediately or stored frozen at −20°C until further processing. When non-specifically aged adult worker bees (foragers) were needed, individuals entering or leaving the hive were collected from the hive entrance. For experiments requiring bees at different ages, eclosed bees were marked with coloured rubber rings (0.120 Mini-Ties, NAOL Australia) that we attached to the waist of the bee between the thorax and abdomen (electronic supplementary material A). This method was developed in order to avoid problems associated with more conventional marking methods, such as paints, which could introduce contaminating substances. It also allowed bees to be raised in a natural hive environment. Marked bees were placed back into their maternal hives and were kept in wire mesh cages left overnight and were then released into the hive. Bees with bands could then be resampled at different ages and were stored frozen at −20°C before further processing. Banded bees performed all the typical behaviours associated with worker honeybees, including hive ventilation (electronic supplementary material B) and flight.

### 2.2. Quantitative analysis of iron

Iron levels were determined for bees 0, 5, 10, 15, 20 and 25 days post eclosion using inductively coupled plasma-atomic emission spectroscopy (ICP-AES). A total of 110 bees were collected for each time point, which was divided into whole (*n* = 10) or body parts (antennae *n* = 100 pairs, head *n* = 100, thorax *n* = 100, abdomen *n* = 100). Three replicates were collected from different colonies. Ageing and collection for replicate A was conducted from January to February 2012 and replicates B and C were conducted from April to May 2013. Some attrition in numbers occurred by day 20 (*n* = 67 and 63 for body part replicates A and B, respectively). For day 25, collection included whole (*n* = 6) and body parts (*n* = 40) for replicate A, while no animals were collected at day 25 for replicate B. Sample preparation for ICP-AES was conducted by first washing the bees in phosphate buffered saline (PBS) (pH 7.4) using a sonication bath (3 × 10 min rinses) then a second wash (3 × 5 min rinses) in a 50 ml falcon tube. Excess moisture was removed by oven drying bees overnight at 32°C. Bees were then either left whole or dissected into antennae, head, thorax and abdomen before being refrozen at −20°C and freeze-dried under vacuum at −65°C for 72 h using a VirTis BenchTop 2 K Freeze Dryer system (SP Scientific, Warminster, PA, USA). The pooled dry weights of whole bees or bee segments were then recorded.

Bee samples were acid digested by adding 10 ml of 69% nitric acid to each sample (plus 2 reagent blanks). Each sample was then placed into a graphite block and heated to 95°C until the volume was reduced to 2 ml, followed by the drop-wise addition of 0.5 ml of 30% hydrogen peroxide. This solution was left to further reduce to less than 1 ml and then diluted to 30 ml (10 ml for antennae samples and blanks) with 1% nitric acid. Samples were then transferred to three (or one in the case of the antennae samples and blanks) 10 ml disposable plastic tubes for ICP-AES analysis using an Agilent 700 Series ICP-AES (Agilent Technologies, Santa Clara, CA, USA). Total Fe per bee part (TFe) was measured by dividing the TFe determined by ICP-AES by the number of individuals in the sample. From this, Fe concentration per gram of tissue (Fe-conc) was calculated using the average sample dry weights.

All statistical analyses of data were conducted using SPSS for Macintosh. We used analysis of variance (ANOVA) and linear regressions to test for effects of age on body mass and iron concentration.

### 2.3. Perl's Prussian blue staining

The distribution of iron within the honeybee abdomen was determined through histological staining with the Perl's Prussian blue (PB) reaction, which renders ferric iron (Fe^3+^) blue in colour [31]. As such, the stain will non-specifically reveal the presence of a variety of iron oxide and iron hydroxide mineral compounds. Newly eclosed (*n* = 3) and foraging (*n* = 4) workers were collected and anesthetised by cooling at 4°C for 2 h. The sting apparatus, ventriculus, Malpighian tubules and the rectum were dissected from the abdomen, which was separated from the thorax and immediately placed into freshly prepared cold (4°C) glutaraldehyde (GA) (2.5%) and paraformaldehyde (PFA) (4%) fixative buffered in PBS (pH 7.4) on a benchtop rocker overnight. A single forager was left unstained as a control, while all other samples were rinsed in fresh PBS before staining for 1 h with freshly prepared PB. The PB stain was made to 5% by mixing equal parts of HCl and potassium ferrocyanide (Sigma-Aldrich) each made to 10% with deionized water. Stained samples were then rinsed with PBS (4 × 1 h) and then dehydrated through a graded series of EtOH (30, 50, 70 and 100% × 2) with approximately 6 h between each step. The dorsal, ventral, left and right sides of each abdomen were then imaged using a Discovery V8 stereo dissecting microscope (Zeiss, Oberkochen, Germany) fitted with a digital camera.

### 2.4. Magnetic resonance and X-ray imaging

For magnetic resonance imaging (MRI), newly eclosed and 25 day old bee abdomens were fixed using cold 4% PFA buffered in PBS (pH 7.4) overnight. Samples were placed under vacuum to minimize trapped air pockets and then mounted in 5 mm diameter tubing filled with PBS. Three newly eclosed and three 25 day abdomens were imaged using a 16.4T MRI (Ultrashield Plus 700 WB Advance NMR spectrometer, Bruker Optics, Billerica, MA, USA) fitted with a quadrature micro-imaging coil. A gradient echo FLASH sequence was used to obtain high-resolution (15–20 μm isotropic) three-dimensional images of the bee parts suitable for qualitative anatomic characterization and segmentation. A multi-echo RAREVTR sequence was used to acquire spin echo data in two-dimensional slices (in-plane resolution 50 µm) from which R2 relaxation rates were calculated. R2 values were calculated for each voxel using the MRI Analysis Calculator plug-in for ImageJ (v. 1.42) (for detailed acquisition parameters see electronic supplementary material C). The software fits a monoexponential decay function of the form, $S(\text{TE})\;=\;S(0){\text{e}}_{2}^{-R*\mathrm{TE}}$ to the data, where *S*(0) is the initial amplitude and TE is the echo time. Voxels were excluded where the goodness of fit parameter was less than 0.9.

For X-ray imaging, adult forager abdomens (*n* = 3) were fixed in cold 4% PFA buffered in PBS (pH 7.4) overnight before dehydrating in a graded series of ethanol to 70% (30, 50, 70%). To increase X-ray absorption in soft tissues, abdomens were heavy metal stained for 24 h using 1% iodine in 70% ethanol (I2E). Samples were rinsed (×3) and imaged in 70% ethanol prior to imaging. Abdomens were scanned at 40 kV and 74 µA using an X-ray micro-computed tomography (X-ray µCT) system (Versa 520, Zeiss, Pleasanton, CA, USA) running Scout and Scan software (v. 10.6.2005.12038, Zeiss). A total of 3201 projections were collected over 360°, each with a 5 s exposure. 2× binning was used to achieve a suitable signal to noise ratio and 0.4× optical magnification was used to achieve an isotropic voxel resolution of 7.9 µm. Raw data were reconstructed using XMReconstructor software (v. 10.7.3679.13921, Zeiss) following a standard centre shift and beam hardening correction of −0.25 and 0, respectively. The standard 0.7 kernel size recon filter setting was also used.

The visualization and analysis of data generated from both MRI and X-ray µCT scans was performed following manual segmentation using Avizo (v. 8.1.1, FEI) software. All measurements were conducted using standard Avizo tools (for workflow see electronic supplementary material D).

### 2.5. Scanning electron microscopy and microanalysis

A single eclosed and 25 day old abdomen was selected from those imaged using MRI and prepared for scanning electron microscopy (SEM) and X-ray microanalysis (i.e. energy dispersive spectroscopy, EDS). Abdomens were dehydrated using a graded series of EtOH as described for PB staining and then infiltrated with epoxy resin (Procure 812, Proscitech) mixed with 100% EtOH at ratios of 1 : 3, 1 : 1, 3 : 1 and then 2× 100% resin, for approximately 24 h each on a rotator. Samples were then mounted in plastic embedding moulds filled with resin and polymerized at 70°C for 24 h.

Sagittal sections through each abdomen were obtained by cutting samples from the resin blocks using a hacksaw and removing excess resin/tissue with silicon carbide grinding paper (1000, 2000, 4000 grit). Final orientations were obtained by mounting the samples in a microtome (EM UC6, Leica Microsystems) and trimming away resin with a glass knife. Block surfaces were polished using a diamond knife (45° Histo, Diatome), then mounted onto aluminium stubs with carbon paste and coated with 20 nm carbon with an evaporative coater (Speedivac 12E6, Edwards High Vacuum Ltd, UK).

For SEM imaging and EDS analysis, samples were examined in a field emission SEM (1555VP, Zeiss) fitted with an X-ray analytical system (Oxford Instruments, X-Max 80 SDD) with thin window detector. For EDS, quantitative spectral data and element maps were collected at an accelerating voltage of 15 kV in high current mode at working distance of 9 mm. Immediately prior to map acquisition, the EDS system was calibrated using a Cu standard and the beam current was recorded. Elemental maps comprised a greater than 400 frame acquisition and a dwell time of 50–100 µs per pixel. Data were processed using Aztec 2.0 software (Oxford Instruments, Oxfordshire, UK).

## 3. Results

### 3.1. Honeybee bodyweight

**Figure 1.**
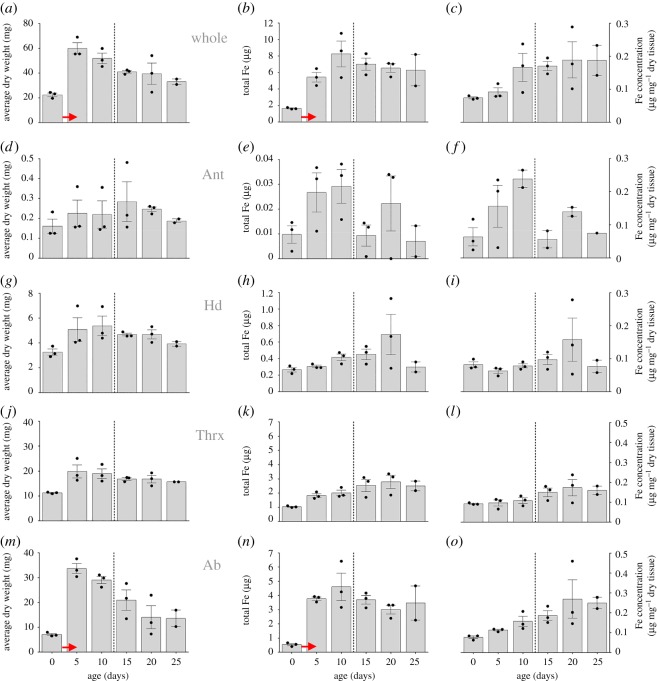
Dry weights, total iron and iron concentrations of adult honeybees and body parts (Ant, antennae; Hd, head; Thrx, thorax and Ab, abdomen) at eclosion and at 5 day intervals to an age of 25 days. Dashed lines represent the approximate start of foraging activities of worker bees. Bar graph data show the average of the three colony replicates ± s.e.m. and dots represent the value for each individual colony replicate as outlined in the methods. Red arrows denote the most significant changes in weight or TFe.

We found that the dry weight of workers significantly differed between age classes (Kruskal Wallis *K* = 13.392, *N* = 17, *p* = 0.020, figure 1*a*) and increased close to threefold during the first 5 days post emergence from 22.45 ± 1.43 mg to 59.98 ± 4.51 mg (means ± s.e.m.). Body weight declined significantly in individuals older than 5 days (linear regression, *F*_1,12_ = 18.746, *p* = 0.001, *r*^2^ = 0.610). When we separately quantified dry weight changes for antennae, heads, thoraces and abdomens (figure 1*d*,*g*,*j*,*m*), we found that thorax and abdomen were the main contributors for the body mass changes observed in whole individuals (figure 1*a*) but the abdomen showed the largest increase in body mass from 7.059 ± 0.454 mg to 33.651 ± 2.094 mg (means ± s.e.m.).

### 3.2. Total iron and iron concentration in honeybee workers

We found that worker age was a significant predictor for total iron (ANOVA, *F*_1,16_ = 5.782, *p* = 0.030) and iron concentration in entire bees (ANOVA, *F*_1,16_ = 11.321, *p* = 0.004). The total amount of iron in bees significantly increased with age (figure 1*b*, linear regression, *F*_1,15_ = 5.084, *p* = 0.030) from 1.65 ± 0.06 µg (mean ± s.e.m.) in newly eclosed individuals to 6.28 ± 2.69 µg in 25 day old workers but most of the iron accumulation occurred during the first 5 days post eclosion (figure 1*b*) and decreased afterwards in animals older than 15 days (figure 1*b*), albeit this was not statistically significant (linear regression, *F*_1,7_ = 0.342, *p* = 0.580 n.s.). The concentration of iron in workers also increased with worker age from 0.07 µg mg^−1^ in animals at eclosion to 0.19 µg mg^−1^ in 25 day old workers (figure 1*c*, linear regression, *F*_1,15_ = 11.321, *p* = 0.004).

When we analysed age-related changes of iron levels in the different body parts, we found significant effects of age and body part on total iron (see table 1 for statistical details). We found the lowest amounts of total iron in the antennae (figure 1*e*), followed by the head (figure 1*h*), thorax (figure 1*k*) and abdomen (figure 1*n*). When we analysed iron concentration between different body parts and ages, we found a significant body part × age interaction term (see table 2 for statistical details), indicating that iron concentrations differed between body parts, but that these differences were specific to certain ages. Similar to our findings on total iron, most significant changes in iron concentration occurred early in the life of workers up to an age of 10 days but did generally not change in any substantial way in older bees (figure 1). Finally, our analysis of individual body parts revealed that the age-related changes of absolute total iron and iron concentration observed in whole animals (figure 1*b*,*c*) were driven by a single body part, being the abdomen (figure 1*n*,*o*).

**Table 1.** ANOVA table summarizing statistical details of age-related changes on total iron levels and body part in the honeybee *Apis mellifera*.

source	type III sum of squares	d.f.	mean square	*F*	sig.
corrected model	121.534^a^	7	17.362	27.736	<0.001
intercept	21.843	1	21.843	34.894	<0.001
body part	18.531	3	6.177	9.868	<0.001
age	6.241	1	6.241	9.970	0.002
body part × age	4.753	3	1.584	2.531	0.066
error	37.558	60	0.626		
total	296.962	68			
corrected total	159.092	67			

^a^*R*^2^ = 0.764 (adjusted *R*^2^ = 0.736).

**Table 2.** ANOVA table summarizing statistical details of age-related changes on iron concentration and body part in the honeybee *Apis mellifera*.

source	type III sum of squares	d.f.	mean square	*F*	sig.
corrected model	0.166^a^	7	0.024	5.729	<0.001
intercept	0.176	1	0.176	42.322	<0.001
body part	0.010	3	0.003	0.804	0.496
age	0.037	1	0.037	8.953	0.004
body part × age	0.064	3	0.021	5.123	0.003
error	0.249	60	0.004		
total	1.431	68			
corrected total	0.415	67			

^a^*R*^2^ = 0.401 (adjusted *R*^2^ = 0.331).

### 3.3. Imaging and analysis of iron in the honeybee abdomen

**Figure 2.**
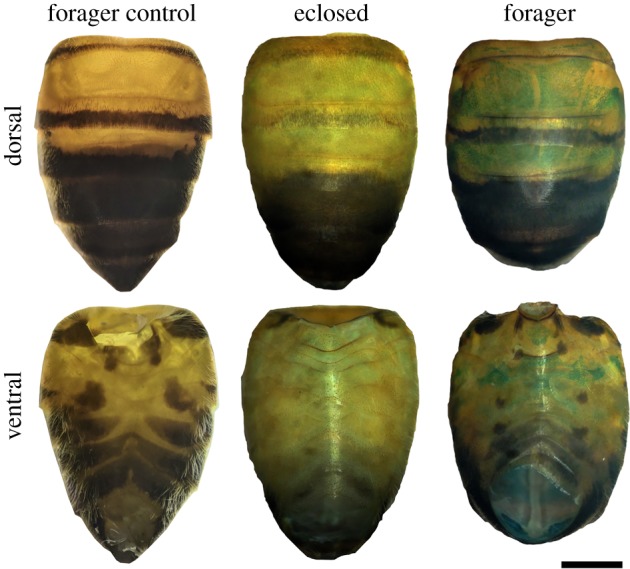
Light micrographs of honeybee abdomens showing an unstained forager (forager control) and a newly eclosed and forager abdomen stained with 5% Perl's Prussian blue. Scale bar, 1 mm.

**Figure 3.**
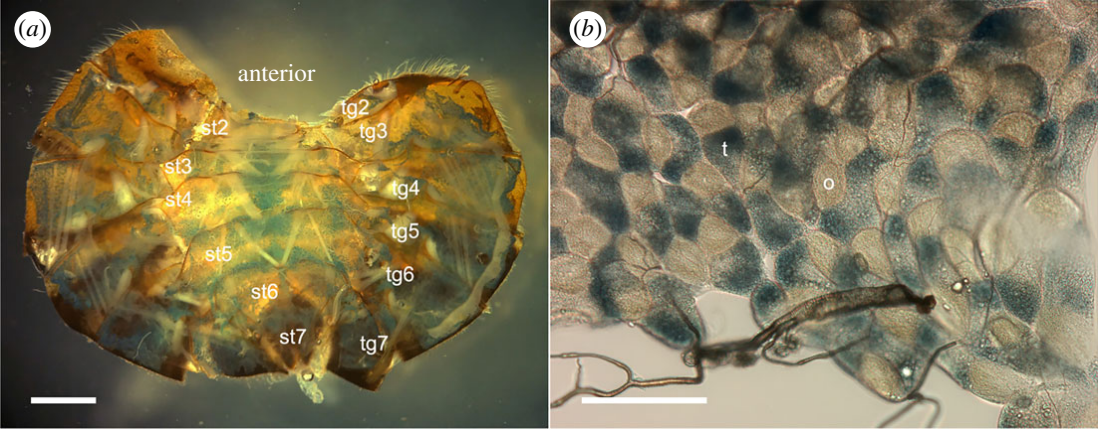
Light micrographs of the honeybee fat body showing the distribution of Perl's Prussian blue stain across (*a*) tergite (tg) and stergite (st) segments in a dissected honeybee abdomen and (*b*) within a sheet of trophocyte (t) and onyocyte (o) fat body cells from tg3. Scale bars, (*a*) 1 mm and (*b*) 100 µm.

Relative to unstained forager abdomens, newly eclosed honeybee worker abdomens exhibit weak staining with Perl's Prussian blue (PB), while foragers exhibit comparatively strong PB staining (figure 2). In foragers, the extent of this PB-stained region is partly obscured by dark cuticle pigment in tergites 4–7 and to a lesser degree by the stergites. The pattern of staining in each of the abdominal segments was similar between individuals (see electronic supplementary material E1–E2). PB staining is evident across much of the dorsal surface of the tergite segments and is less evident on the left and right sides. In the stergites, PB staining is most prominent along the central region of each segment (figures 2 and 3). Staining in tergite 2 is limited to a band of cells at the posterior margin of this segment. In dissected forager abdomens, PB staining is evident in all tergite and stergite segments (figure 3*a*). At higher magnification, the fat body is shown to comprise a patchwork of PB-stained trophocyte cells and unstained onyocyte cells (figure 3*b*).

### 3.4. Magnetic resonance and X-ray imaging

**Figure 4.**
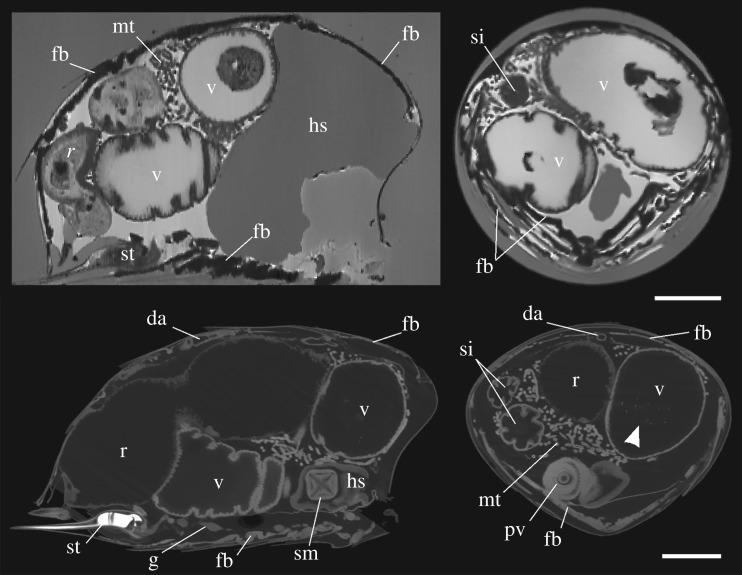
Near sagittal (left) and transverse (right) cross-sections of forager honeybee abdomens imaged using MRI (top) and X-ray µCT (bottom). The internal anatomy is clearly visible, including the fat body (fb), which in MRI imaging appears as a dark hypointense layer below the cuticle, probably owing to the presence of iron in these tissues. Arrowhead denotes bright particulates within the ventriculus. da, dorsal aorta; g, ganglion; hs, honey stomach; mt, malpighian tubules; pv, proventriculus; r, rectum; si, small intestine; sm, stomach mouth; st, stinger; v, ventriculus. Scale bars, 1 mm.

Both MRI and X-ray µCT imaging result in volumetric data that can be ‘virtually’ dissected in any orientation. Figure 4 shows orthogonal slices through the abdomen of a foraging bee as observed by MRI and X-ray µCT. All organs of the alimentary canal and associated glands are visible in our scans, including the stomach mouth, proventriculus, ventriculus, small intestine and malpighian tubules. Notably, X-ray µCT revealed the presence of X-ray absorbing material within the ventriculus in I2E-stained (figure 4) and non-I2E-stained samples (data not shown). Other anatomical features such as the sting, ventral nerve ganglion and dorsal aorta are also visible.

**Figure 5.**
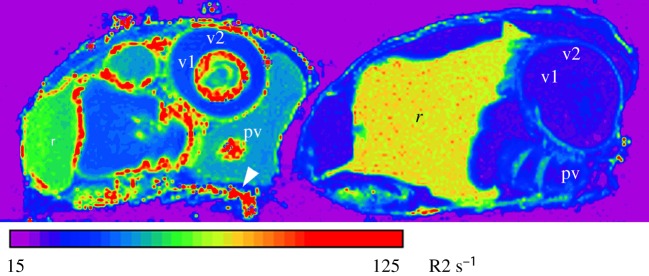
R2 map of sagittal slices through the abdomen of a forager (left) and an eclosed (right) adult honeybee, scanned side by side. Note the high R2 signature (hot colours) in the forager ventral fat bodies (arrowhead), the proventriculus (pv), the inner and outer epithelial layers of the ventriculus (v1 and v2, respectively), which are largely absent in the eclosed bee. Notably, the region corresponding to the rectum (r) in the eclosed bee is enlarged and has a higher R2 signature compared to the forager.

Quantitative R2 relaxometry (figure 5), which is related to the concentration of magnetic species in tissue, illustrates the dramatic changes in the bee abdomen with age. In general, the transverse relaxation rate is higher in the forager bee across nearly all regions and organs. In particular, the mean transverse relaxation rate of the iron-rich layers is on average 62% higher (*n* = 3, *t* = 9.0015, *p* = 0.0001) in foragers compared to eclosed bees. The inner and outer epithelial layers of the ventriculus in the forager also display very high R2 values and are on average 102% (*n* = 3, *t* = 4.9019, *p* = 0.0001) and 133% (*n* = 3, *t* = 10.782, *p* = 0.0001) higher in foragers compared to eclosed bees. These changes in relaxation rates with age are consistent with the increased iron concentration observed in the abdomen of older bees as determined by ICP-AES (figure 1) and the enhanced PB staining of the foraging bees compared to the eclosed bees (figure 2).

The fat body is visible in unstained MRI and I2E-stained X-ray µCT abdomen samples (figures 4 and 5), presenting as an almost continuous layer of cells sitting just below the interior boundary of the cuticle. The distribution of the fat body is consistent with the pattern observed in PB-stained abdomen. The fat body in tergite segments is separated along the midline by the dorsal aorta. For MRI imaging the fat body appears as dark material, which we attribute to the presence of iron. The presence of magnetic iron-containing material, in combination with the high magnetic field strength and gradient echo sequence used for imaging, will produce strong localized decay of the MRI signal and the resulting hypointense signature seen in figure 4. Hence, these dark bands are thought to be the iron-rich trophocyte cells, similar in scale and structure to those depicted by [32]. These dark layers become especially thick along the ventral midline, consistent with the PB staining.

**Figure 6.**
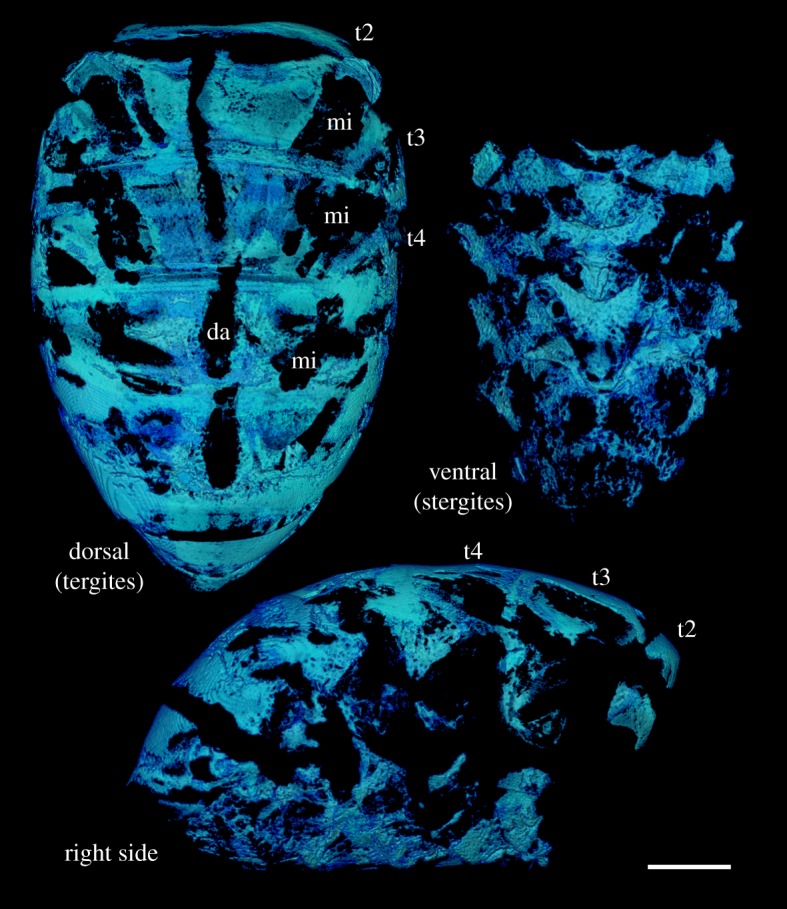
Three-dimensionally rendered visualizations of a forager honeybee fat body segmented from the same X-ray µCT data as shown in figure 4. Segmentation of the fat body is aided by the enhanced contrast from I2E staining and the presence of highly X-ray attenuating material such as iron. However, other strongly I2E-stained features, such as muscle insertion (mi) points and the dorsal aorta (da), complicate the digital segmentation process and the ability to separate the fat body from these tissues. Tergites 2–4 have been labelled for reference. Scale bar, 1 mm.

Using the X-ray µCT data shown in figure 4, the fat body was segmented from the dataset and visualized (figure 6). In tergites 2, 3 and 4, the fat body distribution matches well with the pattern of PB staining of forager abdomens shown in figure 2. The surface area of the abdominal cuticle and the abdominal volume was calculated as approximately 95 mm^2^ and approximately 58 mm^3^, respectively. Using the segmented fat body as a guide (figure 6), it is estimated that the surface area of the fat body covers approximately 65% of the cuticle area. The fat body volume was calculated as 1.67 mm^3^, which was further divided into the volume of the fat body in the dorsal (tergite) and ventral (stergite) regions of the abdomen as 1.23 mm^3^ and 0.44 mm^3^, respectively.

**Figure 7.**
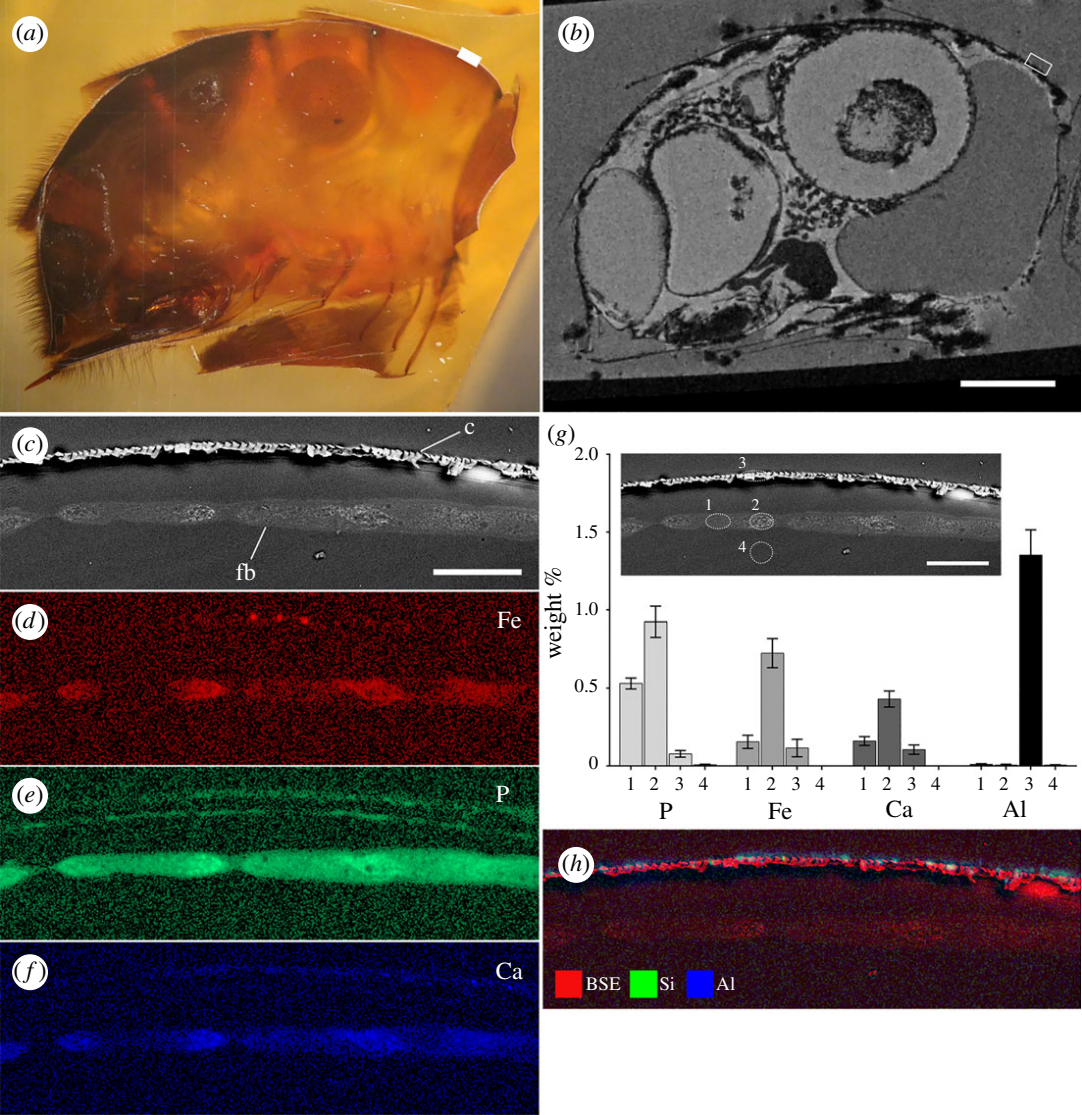
Semi-correlated light (*a*) and MRI (*b*) micrographs of the same forager honeybee showing the abdomen in sagittal section. The boxed region denotes the area on tergite 3 chosen for SEM imaging and analysis (*c–h*). Electron micrographs (*c* and inset *g*) and EDS elemental maps (*d–f*) of the honeybee fat body (fb) in sagittal section below the cuticle (c) of tergite 3. The back-scattered electron (BSE) image (*c*) highlights the presence of electron dense inclusions within the cells that correspond to (*d*) iron (Fe), (*e*) phosphorus (P) and (*f*) calcium (Ca) in the EDS maps. Quantitative EDS taken from four locations (inset *g*) show the amount of each respective element in weight % within these tissues. Note that the major elements present (such as carbon, oxygen and hydrogen, which are present in epoxy resin) are not shown. Si and Al were also observed on the cuticle surface (*h*). Scale bars, *a* and *b* = 1 mm, *c*–*h* = 50 µm.

In order to validate the presence of iron within the hypointense region observed for the fat layer in MR-imaged samples, a single MRI sample was prepared for SEM X-ray microanalysis (EDS). The abdomen was sectioned to expose a plane in a near sagittal orientation (figure 7*a*). Features in the optical and SEM images were then matched as closely as possible to the same orthogonal slice in the MRI data (figure 7*b*). A region of the fat body below the cuticle in tergite 3 was then chosen for EDS. Back-scattered imaging of this region clearly shows the cuticle and fat body within this region (figure 7*c*). The cuticle appears as a layer of fractured looking material owing to the poor infiltration of resin. Elemental mapping of this same region revealed the presence of iron, phosphorus and calcium within the fat body, with higher signal intensities corresponding to the brighter features observed in the back-scattered image (figure 7*d–f*). Quantitative EDS was undertaken using the spectral data for four tissue types (repeated in four similar regions), including the fat body (figure 7*g*, locations 1 and 2), the cuticle (figure 7*g*, location 3) and below the fat body (figure 7*g*, location 4). The main elements present in these tissues (separate from those associated with epoxy embedding media) were P, Fe and Ca (figure 7*g*). A number of elements were also detected on the cuticle surface, with co-localized Al and Si being the most dominant (figure 7*h*).

## 4. Discussion

A number of studies have now provided behavioural and physical evidence implicating the honeybee abdomen as the location of an iron-based magnetoreceptor system, with various superparamagnetic (SPM) and/or single domain (SD) magnetite particles having been described using direct imaging and indirect magnetometry techniques (reviewed by [2]). By undertaking a detailed analysis of iron accumulation in honeybees reared under natural hive conditions we have confirmed that the bulk of the iron is located within the abdomen and accumulates rapidly upon emerging from the comb. It is curious that the abdominal total iron complement achieves and maintains such high levels only 5 days post eclosion, which is well before the commencement of activities outside the hive when a magnetic sense would be necessary. Given directional information for foraging site locations is passed to other hive mates via the waggle dance [33], a phenomenon previously linked to magnetic cues [14], the early development of a magnetic sense in young workers may allow them to read these cues from hive mates.

The rapid accumulation of abdominal iron observed here is consistent with observations using proton-induced X-ray emission (PIXE) to assess iron levels in the fat body of laboratory reared honeybees [32]. The PIXE analysis revealed that iron in the fat body of bees fed on both iron-rich and iron-deficient diets reached similar levels to wild foraging bees well before the commencement of foraging activities. Additionally, the number of iron granules per trophocyte cell plateaued after only 9 days post eclosion. While it is generally thought that fat body iron accumulates in response to an iron-rich pollen diet [30,34,35], the rapid and early saturation of iron within the fat body has led some to speculate that iron accumulation in this tissue is a ‘directed action’ for a purpose other than a response to storing excess iron from dietary sources [32].

The analysis of iron in whole animals and separate body parts has revealed the relative distribution of iron in these segments as they transition from young to mature foraging adults. The ability to trace cohorts of specifically aged bees within the hive environment was made possible using a novel banding technique that ensured natural hive behaviours and avoided the introduction of contaminants to the elemental analysis. A number of observations were made that can be linked to the known behaviour and life history of the honeybee. In the first 5 days, bees gained a substantial amount of weight, which was primarily driven by gains in the thorax and abdomen. This is likely to be attributed primarily to feeding on pollen, but the development of various glands is also likely to have contributed to the observed increase in weight [36]. For example, the increase in head weight at day 5 is probably due to the development of the hypopharyngeal glands in preparation for the secretion of brood food [37].

The correlative MR and SEM imaging combined with EDS analysis of the anterior dorsal region of the abdomen verified the presence of iron, calcium and phosphorus in the fat body. This finding is consistent with previous reports on the composition of iron granules within the fat body [23,24,28,29,32,35]. Perl's Prussian blue-stained abdomens, together with X-ray µCT and MRI, revealed that the iron-loaded fat body exists as an almost continuous sheet of tissue beneath the cuticle, covering approximately 65% of the cuticle area. The fat body is present in both the tergites and stergites, and hence the fat body in the stergite segments is proportionally smaller than that of the tergites. In addition to claims of an iron-based magnetoreceptor located within the anterior dorsal region of the abdomen [17,21,26], the ventral fat body has also been marked as a potential area of interest [22,28]. However, the iron in the trophocyte cell granules is reported to be the relatively disordered and weakly magnetic form of iron oxide ferrihydrite, as found in the iron storage protein ferritin [23], which is not considered to possess sufficient magnetism to act as a torque-based magnetic particle receptor system (i.e. the magnetite hypothesis) [1,38]. However, the recent discovery of a ferrihydrite iron organelle (cuticulosome) within the cuticular plate of avian inner ear hair cells provides a precedent for a non-magnetite-based iron oxide with postulated links to magnetoreception [3,39]. Additionally, the existence of a more ordered form of ferrihydrite that develops ferrimagnetic properties with ageing has also been verified [40]. Could the early and rapid production of iron granules within the trophocyte cells lead to a more mature form of ferrihydrite with enhanced magnetic properties? While some organisms exert an extraordinary level of control over iron biomineralization, which could make such a mineral phase conceptually feasible, the high levels of phosphate reported in iron granules are likely to prevent the crystallization of more ordered iron oxide phases [23], which argues against a ferrimagnetic phase of ferrihydrite within the honeybee fat body.

In MR imaging the fat body appeared as a dark hypointense layer below the cuticle and appears far thicker in the three-dimensional ISO images compared to that shown by X-ray µCT. While MRI does not have sufficient resolution to image individual iron granules, the magnetic influence such particles have over the proton relaxation of surrounding tissue may lead to visible susceptibility effects far larger than the particle itself [41]. As MRI samples were scanned in a hydrated state, versus X-ray µCT and SEM, which were dehydrated using ethanol, it is not possible to make a direct comparison of the thickness of the fat body in MRI and X-ray µCT/SEM data. However, it is likely that the large amounts of iron in the fat body have generated this enhanced susceptibility and apparent thickening of the tissue in this layer. While this property may support the use of MRI in searching for magnetoreceptors in animals, its utility in probing the honeybee abdomen may be limited as the fat body iron could mask other signals in this region, such as those reported to exist within the anterior dorsal region of the abdomen [17,21,26].

The use of SEM imaging and EDS analysis on the anterior dorsal region of the abdomen also revealed the presence of various elements, including iron, aluminium and silica, on/within the honeybee cuticle. While this represents only a single cross-section through a single animal it does raise the issue of exogenous sources of contamination when searching for magnetoreceptors in animal systems. This is further highlighted by the small X-ray opaque features observed within the ventriculus of the honeybees imaged using X-ray µCT, which are likely to be dense particulates (possibly iron) incidentally ingested as part of the diet. Honeybees are known to concentrate impurities from the environment, and have been used extensively as indicators for the presence of various pollutants [42]. To date, studies using direct imaging approaches or indirect magnetometry-based analyses to demonstrate the presence of magnetic material in insects have made little effort to account for these contaminants [2]. Future studies aimed at describing the particle composition of insects should control for the presence of particulates that may be present in the digestive system or on the waxy epicuticle.

A marked difference was observed in quantitative R2 relaxometry between eclosed and foraging honeybees imaged using MRI, and we suggest that this is a reflection of the accumulation of iron as the animal ages. In particular, parts of the digestive system gave strong R2 responses in foraging bees. This is most likely an indication of the iron granules, similar to those in the fat body, present within the ventriculus, which are suggested to play a detoxification role for elements such as iron, manganese, zinc and lead [34]. This is supported by analyses using laser ablation-inductively coupled plasma-mass spectrometry, which also associated these elements with the alimentary canal of honeybees and specifically attributed dietary intake as the primary pathway for environmental pollutants entering the digestive tract [43].

Whether the honeybee fat layer plays some role in magnetoreception or is simply a store of excess dietary iron remains an open question. The data provided here seeks to provide information on the distribution of iron within the honeybee in the context of the animal's life history and abdominal anatomy. It has also highlighted the need to be cautious about endogenous or exogenous sources of iron contamination, which have plagued the field of magnetoreception for many years, as exemplified by the recent corrections observed in pigeons and trout [9,11]. There is an obvious need to employ new investigative techniques that can address the search for rare cell types of unknown location. The multi-modal imaging and analytical approach adopted here seeks to provide some inspiration for future studies, which will undoubtedly require the application of correlative or semi-correlative investigative methods. The use of bulk techniques, such as ICP-AES, have revealed patterns of iron utilization that correspond to aspects of honeybee development and behaviour, while the various imaging methods have exposed potentially useful methods for visualizing iron and the overall anatomy of the animals being studied. Only a combined approach will reveal the true mechanistic basis of magnetoreception.

## Supplementary Material

SupMaterial A-E

## Supplementary Material

SupMaterial B

## Data accessibility

Our data are deposited at doi:10.4225/23/5af0289550860.
