# Supplementary material for: Multi-modal imaging and analysis in the search for iron-based magnetoreceptors in the honeybee Apis mellifera
Source: R Soc Open Sci. 2018 Sep 19;5(9):181163. doi: 10.1098/rsos.181163 (PMC6170574; doi:10.1098/rsos.181163)
Supplement: SupMaterial A-E [file rsos181163supp1.docx]

SupMaterial A


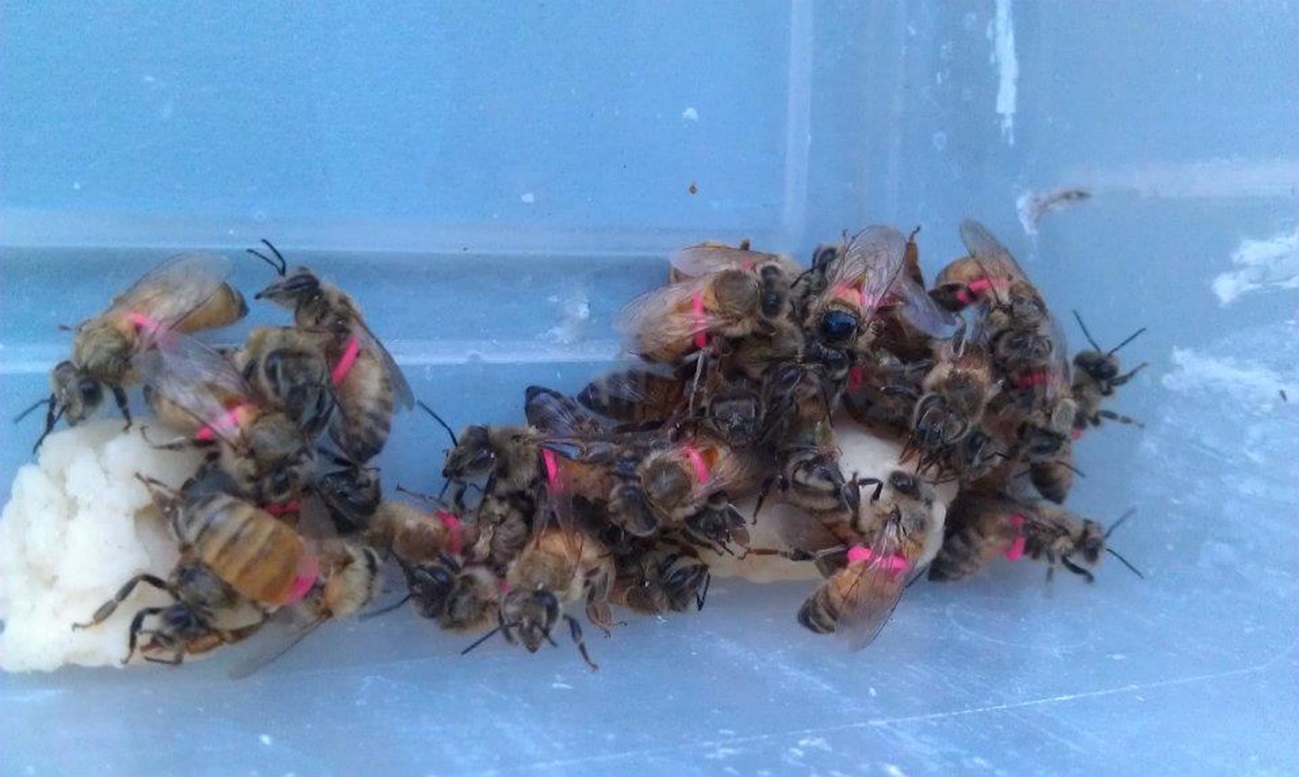


Newly eclosed honey bees banded with coloured rubber rings.

SupMaterial B

Video footage of bee hive entrance with banded bee undertaking ventilation activities.

SupMaterial C

MRI acquisition parameters for 3D high-resolution scans and 2D R2 relaxometry.

| **Parameters** | **Abdomen** | |
| --- | --- | --- |
|  | **3D ISO** | **2D R2** |
| Sequence Name | FLASH | RAREVTR |
| Echo time (ET) (ms) | 3.53 | 7.65 - 61.2 |
| ET Step size (ms)/No. | - | 7.65/8 |
| Repetition time (TR) (ms) | 40 | 4 000 |
| Flip angle (degrees) | 12 | 90, 180 |
| Signal averages | 2 | 8 |
| Matrix dimensions (pixels) | 1068 x 256 | 512 x 104 |
| Field of view (mm) | 25 x 5 | 25 x 5 |
| Slice thickness (µm) | 19.5 | 200 |
| In Plane Pixel Resolution (µm) | 19.5 x 19.5 | 48.8 x 48.1 |

SupMaterial D

Avizo Fire 8.1 Segmentation and analysis workflow

| **Step** | **Avizo module** | **Sub-settings** | **Justification/Purpose** |
| --- | --- | --- | --- |
| 1 | Volume Render and Ortho Slice | Colormap altered as required. | Basic visualisation of data |
| 2 | Extract Subvolume | N/A | Crops dataset to relevant region of interest (ROI) to minimise volume size and computational requirements. |
| 3 | Non-Local Means Filter | Default software settings | Filter: Implements a windowed non-local means algorithm for denoising scalar volume data. |
| 4 | Edit New Label Field | Tools utilised*:  Brush, Magic wand, Thresholding | Activates the Avizo segmentation editor to define labels for different ROIs. |
| 5 | Label Analysis | Interpretation – 3D  Basic measures – including Volume3d and Area3d. | Performs measurements on connected components of the label fields generated at #4. |

* Segmentation in Avizo Fire was undertaken manually using combinations of the tools listed above and taking advantage of other features in the software such as working with locked and unlocked label fields, tools in the selection menu (grow, shrink, fill, invert and interpolate) and the segmentation menu (remove islands and fill holes).

A manual segmentation approach was chosen owing to the highly interconnected nature of the various organs within the honey bee abdomen and the limited grey scale resolution separating these organs in both the X-ray µCT and MRI data.

SupMaterial E1


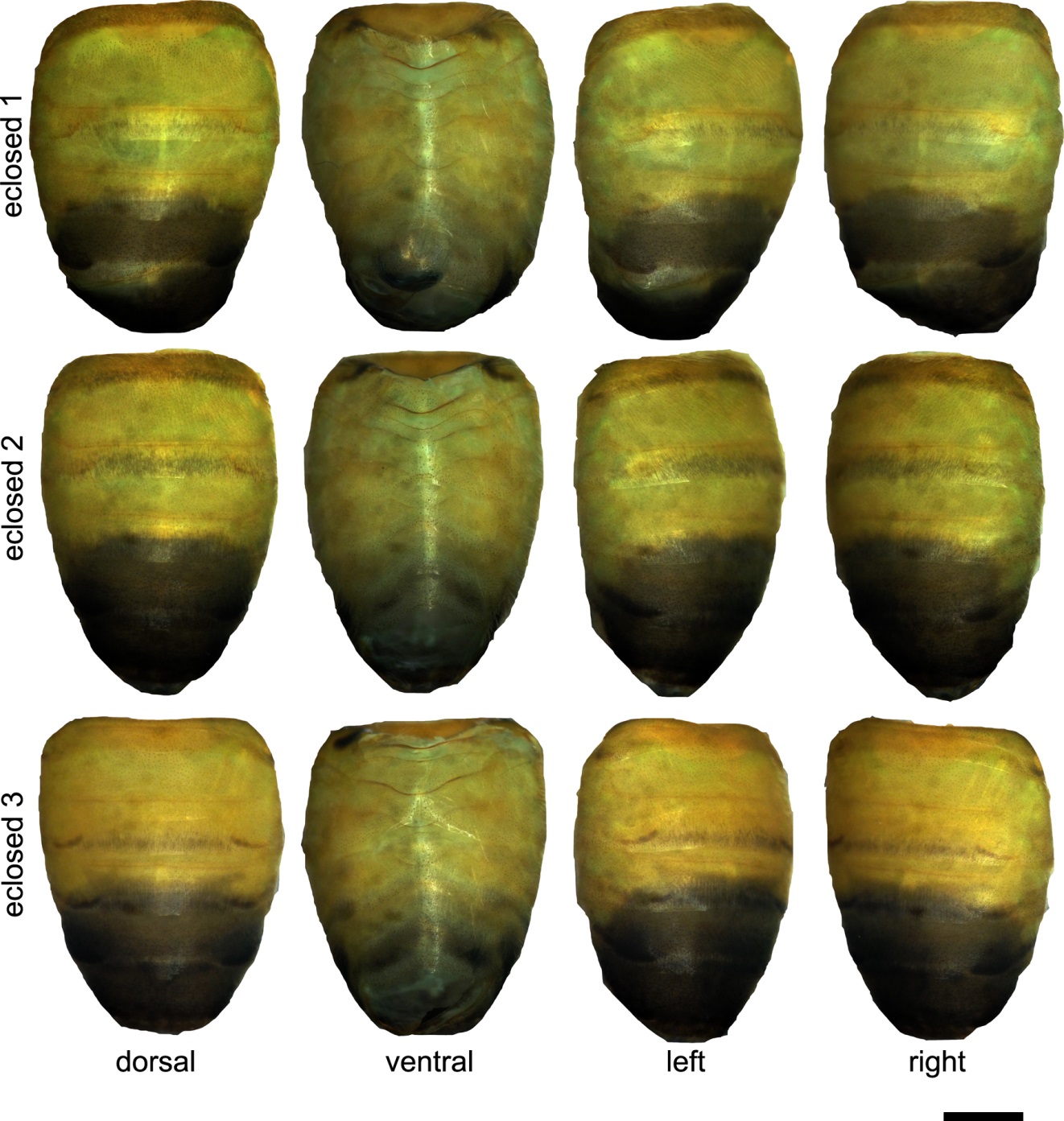


Supplementary Figure E1: Light micrographs of three eclosed honey bee abdomens stained with Perl’s Prussian blue. Scale bar = 1 mm.

SupMaterial E2


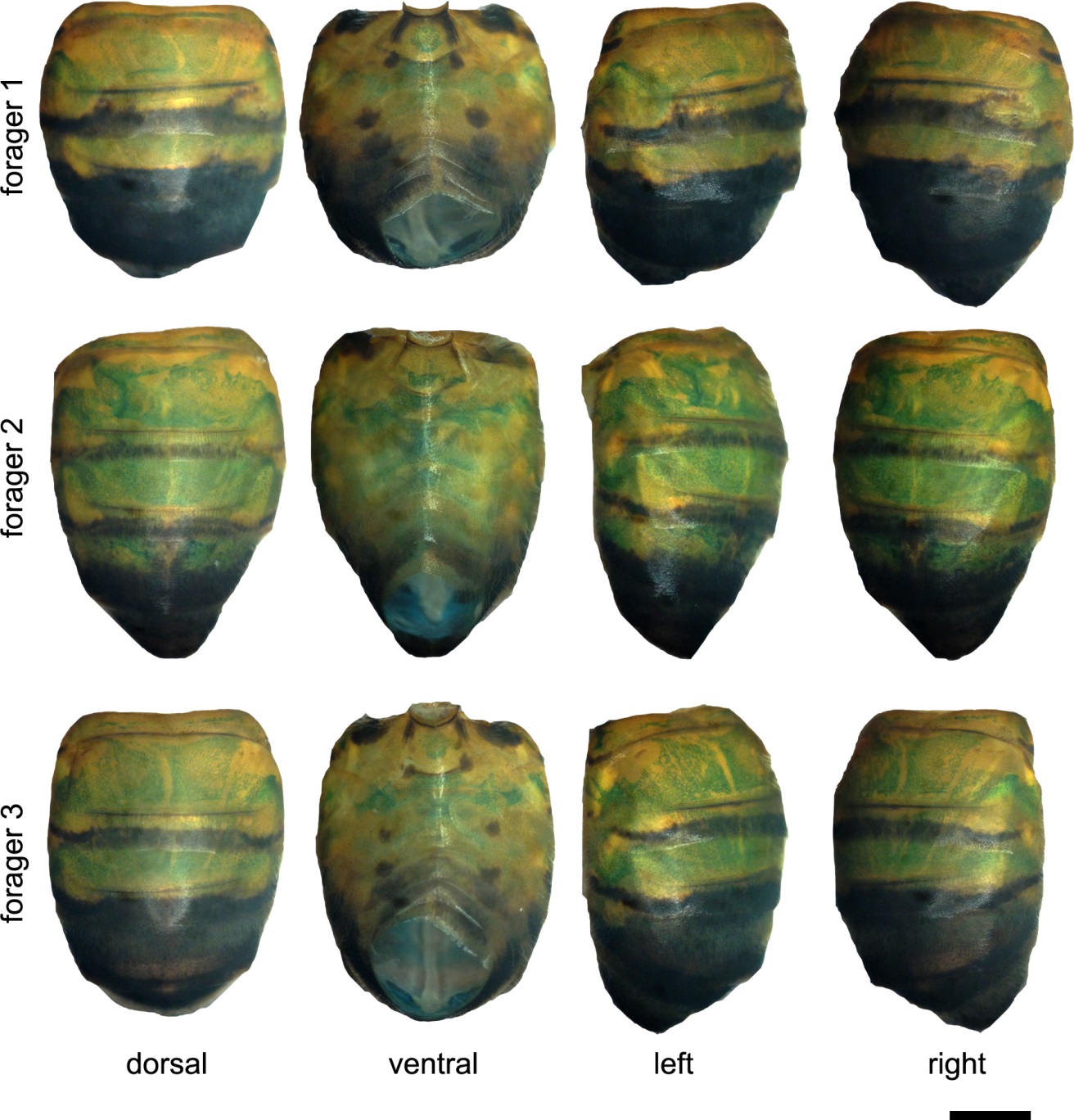


Supplementary Figure E2: Light micrographs of three forager honey bee abdomens stained with Perl’s Prussian blue. Scale bar = 1 mm.
